# Identification of a Workpiece Temperature Compensation Model for Automatic Correction of the Cutting Process

**DOI:** 10.3390/ma15238372

**Published:** 2022-11-24

**Authors:** Anna Zawada-Tomkiewicz, Dariusz Tomkiewicz, Michał Pela

**Affiliations:** 1 Faculty of Mechanical Engineering, Koszalin University of Technology, Śniadeckich Street, 75-453 Koszalin, Poland; 2 D&H Engineering, Perłowa 13 Street, 77-132 Niezabyszewo, Poland

**Keywords:** process correction, workpiece accuracy, SPC, identification, NLARX

## Abstract

This article describes a system for measuring and compensating for errors resulting from the cutting process in order to improve the accuracy of the workpiece. Measurements were performed by means of an automatic measurement unit. The diameter of the workpiece was measured at two points, and at the same time, the temperature at the end face of the workpiece was measured. These measurements were used in Statistical Process Control (SPC). Based on the measured values, the process stability was checked and an error correction value was determined for the next item. Moreover, the value of the correction was influenced by the assumed value of tool wear, in accordance with the adopted model, and the possibility of achieving the assumed surface quality. The diameter of the workpiece for SPC purposes was measured under industrial conditions using an automatic measurement unit, which indicates that the temperature of the workpiece during the measurement was significantly higher than the reference temperature. The study focuses on the possibility of identifying a workpiece temperature compensation model in measurements of the workpiece diameter for the purpose of introducing an additional change in the correction value. It was found that a model with a constant correction value and a linear model poorly reflect the nature of the changes. On the other hand, the Autoregressive with Extra Input (ARX) model and the Nonlinear Autoregressive with Extra Input (NLARX) model, with a neural network, are able to map the inertia of the system and map the process with greater accuracy. In this way, measurements performed in industrial conditions can more accurately determine the possibility of achieving the assumed tolerance of the finished product. At the same time, the research shows that the temperature compensation model is nonlinear, and that the maximum possible machining accuracy of the workpiece can be achieved thanks to the repeatable measurement and compensation technique.

## 1. Introduction

Achieving high accuracy of the workpiece is one of the greatest challenges in industrial manufacturing [1,2,3,4,5]. In the case of production lines with high precision requirements, due to the narrow tolerances of each elementary process, high-specification and high-precision machines are required, resulting in high equipment costs [6]. The accuracy and precision of manufacturing systems can be achieved and improved by real-time measurement and error compensation in the manufacturing process [7,8]. This allows systems to compensate for errors and to improve accuracy and precision in machining. Therefore, the accuracy of machining is achieved thanks to the use of modern, super-accurate solutions to compensate for hardware and software errors. Compensation software for controlling the accuracy of machining is developing along with the availability of high-accuracy computers and sensors.

Technological quality is produced in a process whose impact is force and temperature [9,10,11]. This indicates that the workpiece measured immediately after machining is not temperature-stabilized, and it is believed that this stabilization depends on the material and shape of the workpiece, as well as the broadly defined forcing conditions during the process and the stabilization conditions [12,13]. Therefore, a question arises regarding the reverse problem: How to control the process to obtain the assumed technological quality of the product, which is a function of many factors, such as:

- thermal phenomena related to heat emission in the cutting zone [10,14];
- rigidity of the cutting system, its deflections and vibrations [15,16];
- machining parameters, type of tool and machining method, quality of measuring systems and positional repeatability [17,18].

Analysis of the above factors, their identification and partial compensation are the basic elements in improving the accuracy of machining through software process correction [19,20]. The difficulty in developing a uniform compensation system for all machining errors is due to the fact that only some of them depend on the machining system [21,22,23]. The technological quality of the workpiece can be ensured under conditions of narrow error tolerance for the workpiece, for which:

1. Geometric errors of the machining system are compensated by software, and their accuracy is periodically verified using high-accuracy sensors and measurement methods [8].
2. Errors resulting from the mutual displacement of the tool and the workpiece are modeled and then compensated due to the elastic return of the material under the influence of the applied force [9].
3. Errors originating in tool wear are modeled and then compensated in the machining program [11]. Tool wear is the cause of some discrepancies in the modeled and actual characteristics of machining processes, due to nonlinearity.
4. Errors resulting from thermal deformations in the machining process are modeled and compensated in the values of cutting parameters [10,24].

As can be seen from the above, precise error modeling is a critical part of the elimination of errors. The most cost-effective way to obtain high-quality, high-performance products is through the use of error compensation techniques. Sources of errors related to the machining system and methods of compensating for geometric and thermal errors of the machining system by means of software are recognized research topics [14,25,26].

The modeling of production processes is used to predict the result depending on the selected settings. Machine tool–process simulation is used in most published studies for the analysis of machining stability, as in [27], which reports on the influence of the tool nose radius on the stability of turning operations.

On the other hand, the compensation of errors resulting from the cutting process depends on physical phenomena that are nonlinear and often chaotic, the modeling of which takes place in the conditions of difficult-to-machine materials. For example, thermally induced errors can account for as large of an amount as 70% of the dimensional errors of a workpiece [28].

Therefore, compensation for errors resulting from the cutting process is carried out by monitoring and ongoing control of the production process [29]. The basis of process monitoring and control is the analysis of measurements [30]. Process correction as compensation for machining errors can be performed online, where the measuring system is part of the machining system. The cutting correction process is carried out in a very short time, in order that it can be treated as real-time programming. The measurement unit is sensitive to various physical disturbances (temperatures, forces, deflections, etc.) occurring in the measurement process [31]. On the other hand, offline methods are based on the analysis and modeling of various sources of errors that affect the accuracy of the workpiece. The resulting regression relationship between the driven independent variables and the error variable can provide a basis for calculating the process adjustment. After a process correction is made, the accuracy of the post-processing increases significantly.

The aim of this article is to define a methodology for introducing corrections in Statistical Process Control (SPC) in order to compensate for errors in thermal deformation of the workpiece. The basis of the calculated values is a model that describes the relationship between the measured values of the physical quantities of the workpiece immediately after the process (for high temperature of the workpiece and industrial measurement conditions) and under reference conditions. The Section 2 discusses the research methodology related to the general requirements of the production process; then the automatic measurement unit is defined and the implementation of corrections in the SPC system is presented. The Section 3 presents an example of SPC operation and the identification of a workpiece temperature compensation model. Four models were developed and validated: A model with bias, a model with linear dependence on temperature difference (linear model), the Autoregressive with Extra Input (ARX) model, and the Nonlinear Autoregressive with Extra Input (NLARX) model. The Section 4 summarizes the range of applicability of individual models in industrial practice.

## 2. Materials and Methods

The automatic process correction unit was developed under the assumptions listed below:

1. For the cutting process, a process model has been developed, including a model of the wear of the tools used and the achievement of the assumed surface accuracy.
2. An automatic measurement unit measuring the workpiece diameter in industrial conditions has been developed.
3. A procedure for determining the stability of the process and ensuring the achievement of the assumed accuracy of the final product has been developed.

### 2.1. Technological Machining Process

The research was carried out in industrial conditions for an optimized production process. A roadmap for an optimized cutting process consists of a definition of the material and process and product requirements; next, the model of the process structure and behavior is developed; and finally the constraints and parameters of the process are specified [32].

**Figure 1:**
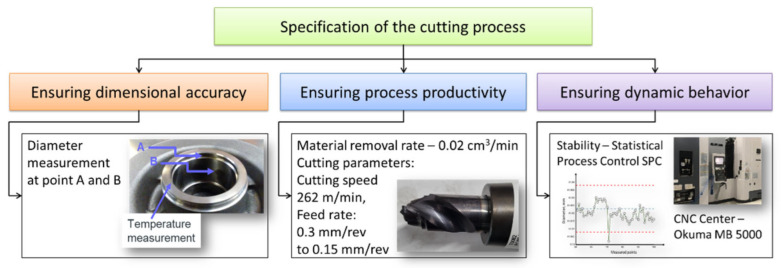
Requirements for the cutting process.

When designing the cutting process, the most important requirement taken into account was the quality of the product. The workpiece has a complex shape with many measured physical quantities (measurands). In this study, quality was considered in terms of the dimensional accuracy of the workpiece diameter, which was measured at two points. The geometric accuracy is influenced by many features, which are presented in Figure 1. These features are related to each other. It follows that obtaining the assumed precision parameters requires, among other things, a suitably stabilized process.

Therefore, the first step in achieving the desired quality of the product in certain production situations is to specify the goal of production and then ensure the quality by considering all of the predefined requirements of the cutting system [2]. These requirements are important since the production environment changes dynamically, and control dependencies between product and production parameters cannot be described analytically.

Product quality generally correlates with many process variables. Model-based self-optimization requires sufficient data regarding these variables, in respect of both quality and distribution, to be able to describe the conditions at the operating point with adequate precision [33].

The basic variable in the process is the material and its parameters. For the cast iron used in the research, the chemical composition, microstructure, mechanical properties of casting, and physical properties were determined (Table 1).

To maintain targets in product tolerance, the values of the process variables must also be kept within predefined limits. The main process variables are the behavior of the workpiece during cutting, the tool used, machine behavior, and the cutting speed and feed rate.

Cutting speed and feed rate can be read directly from the machine control. For the special tool designated as T0082, the rotational speed was selected in the amount of 1350 rev/min, for the nominal diameter it was 262 m/min. The feed was variable from 0.3 to 0.15 mm/rev. Nevertheless, this information is not sufficient for process optimization, which is why further sensors are used. The sensors need to measure additional process variables, and it is essential that they be integrated into the manufacturing system to enable self-optimization [1].

The general requirement of the cutting process is workpiece accuracy. This accuracy can be achieved; however, it is fundamentally influenced by all of the elements in the system. This indicates that all elements must be designed having in mind the achievement of the prescribed accuracy of the workpiece. Sustainable production causes the cutting process to be directed toward low tolerances, which requires sufficient rigidity of the machine tool and diagnostics for tool wear and the cutting process [11].

The interdependencies between product tolerances and the cutting mechanism are analyzed, and a control loop with adaptation of the control goal must be implemented. In the case of workpiece quality assurance, it is not sufficient to measure the diameter after the process. It is necessary to implement an online system. This will make it possible to intervene in the process and adjust individual values and tolerances in response to deviations in previous steps of the process. In this way, the achieved quality of the product can be made to the greatest extent possible in a certain production situation [21,22].

A model of system behavior refers to use cases of the product and the model of the cutting process, which embrace the whole life cycle of the product.

The sources of the final uncertainty of the workpiece are strictly correlated with the actual cyber-physical model of the cutting process, which is generally geometrically oriented. Geometry in manufacturing is prone to deviations, which imposes on designers the task of specifying suitable dimensions and tolerances for components, in order that the functionality of the product is ensured [34].

It can be clearly seen from the definition of requirements, structure, and the behavior of the model that constant parameters and constraints are not sufficient for implementation of the system. The system should be able to define feedback between system structure and behavior. This implies the need to optimize the model and its parameters.

**Figure 2:**
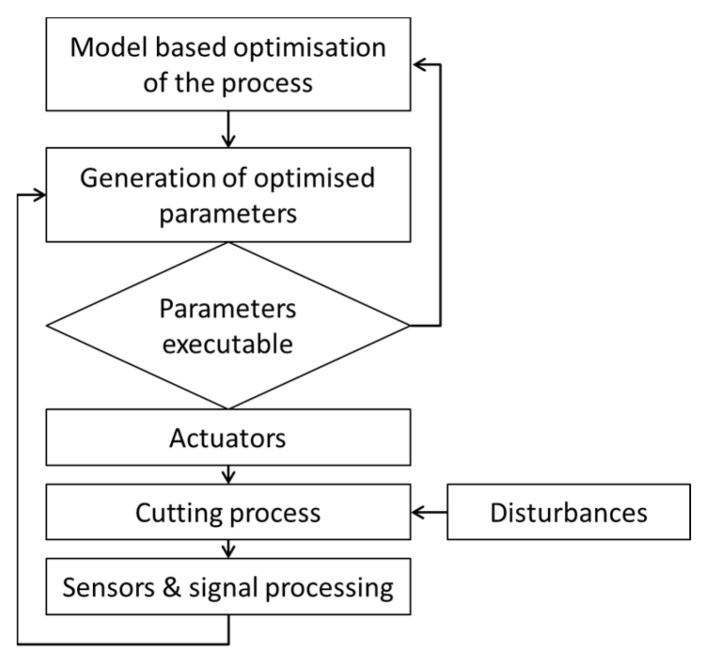
Cutting process optimization.

To adopt a cyber-physical approach, the system must be equipped with data analysis, modeling, and optimization (Figure 2). Once the model of the process is developed, it becomes possible to optimize parameters and generate the process. Parameters which can be used to produce a desired product are then applied in manufacturing. Due to possible disturbances during cutting (tool wear, vibrations), the monitoring system (sensors and signal processing) is applied. Its role is limited to the identification of disturbances and enforcement of process parameter correction [32]. If the changes in parameters are not acceptable in relation to the final product, the model for process optimization is liable to change.

Model-based optimization of the process lays a foundation for generating optimized parameters for the manufacturing system. The parameters are calculated to ensure the quality of the product. Modeling can consider many factors, including those related to process changes. Not all changes are sufficiently predictable to be subjected to modeling; however, the model is optimal provided that the conditions for which it is developed are met. Changing external or internal conditions in the process may lead to inadequacy of the model and its parameters. Therefore, there appears to be a need for cognitive systems to deal with these situations.

To achieve the assumed quality of a product in the cutting process, it is necessary to implement the desired autonomy in the production system [35]. This can be accomplished by means of digital automation of data processing, decision-making tasks, and optimization. The system should be equipped with artificial sensors and actuators, which are integrated and embedded into physical systems and act in the physical world [5].

The main element of the system is cognitive control (behaving in accordance with long-term intentions) and the connection with other elements of the system featuring cognitive capabilities (perception, reasoning, learning, and planning). The cyber-physical cognitive system can reason using substantial amounts of appropriately represented knowledge, learns from its experience, and is aware of its own capabilities.

### 2.2. Automatic Measurement Unit

In the case of the machining process, measurement during the actual machining process without interrupting that process is only possible if the measurements do not directly relate to the workpiece. The reason is that there are disturbances in the cutting operations (chips and coolant). For indirect measurements, the solution enables real-time measurement, and the information generated by in-process measurements is provided continuously. An example of indirect measurements may be the measurement of cutting resistance or vibrations.

A review of the existing measurement systems and their software enabling measurement on the production line did not reveal the existence of ready-made devices in the form of automatic measurement units. Therefore, this unit was developed. Its basic functionality relates to automatic operation that does not require the presence of an operator. It is a unit developed within the organization to carry out measurements on the production line in cooperation with an industrial robot. The measurement can be performed directly on the machine or off the machine, but is performed during the process cycle. The workpiece can be assessed during or after machining.

An automatic measurement unit made it possible to measure the diameter of the workpiece. In the case of diameter measurements, the measuring instrument was a double-contact bore gauge. The diameter was measured at two points labeled A and B, and the temperature of the workpiece face was measured. Measurement point B was at the bottom of the bearing bore, and measurement point A was closer to the start of the bore. The temperature was measured at the face of the workpiece. Measurement of diameter and temperature using the automatic measurement unit was performed within 1 min of the completion of the cutting operation.

The reference measurement was carried out after the workpiece was stabilized to a temperature of 23 °C. Post-process measurement was performed on an independent machine after the workpiece was removed from the process. The advantage of this method is that post-process measurement covers the effects of all error sources that affect the workpiece in one set-up [30]. Compared with in-process measurement, post-process measurement is time-consuming and carries the risk of producing multiple defective items before the inspection results are known.

### 2.3. Implementation of Corrections in the SPC System

The research problem concerns the current correction of the cutting process in industrial conditions, for which the workpiece is characterized by a significantly higher temperature than in the case of a workpiece in a stabilized state. To obtain the maximum possible accuracy for the diameter of the workpiece, a system for measuring and compensating for repeated errors was developed. The procedure for handling the automatic process correction consisted of several stages.

1. Before starting the analysis, all tools were replaced with new ones. Then, the automatic measurement unit was set up and its measuring ability was confirmed. The automatic measurement unit was incorporated into the production cycle. This indicates that all measurements were performed automatically, with full recording of the results. After a correction or a tool change, this information was saved in the database with indication of the correction value and the workpiece number (measurement point). The diameter of the workpiece after the tool position correction was measured by the same method after stabilizing the temperature (reference measurement). The deviation between the expected and measured values was calculated and the nominal toolpath was modified appropriately before the next part was machined. The reference temperature at which the reference measurement was carried out was set at 23 °C.
2. Conduct of a full experiment—all measurements were carried out by the automatic measurement unit. The automatic measurement unit measured the diameter of the hole closer to the face of the workpiece (measurement at point A on the workpiece) and inside the hole (measurement at point B on the workpiece). During the measurements, corrections were made in the process with regard to improvement of the process stability and the wear of the tools. The process correction program takes into account tool wear based on an experiment. Without tool life compensation, producing parts to a given tolerance would mean an increase in tool costs, and in the case of frequent tool changes, there would be no guarantee that the given tolerances would not be exceeded. Tool wear entails shortening of the tool, which causes a constant shift for the entire machined profile. When programming the change in cutting depth, the appropriate measurement error is used to quantify the parameters of the linear approximation. In order to meet the chip forming requirements and the necessary undeformed chip thickness, the process correction value was selected to obtain the minimum cutting layer thickness.
3. Analysis of the measurement data, automatic measurement, and identification of the measurement error were performed in order to adjust the automatic process correction system. Using the process cycle measurement performed by the automatic measurement unit, the value of the physical quantity of the workpiece being machined (workpiece diameter) was measured immediately after cutting. One of the main advantages is the ability to use measurement data to derive corrective actions to improve the accuracy of the machining process (Figure 2). As a result, the performed measurement is used not only to obtain the final assumed value of the physical quantity of the workpiece, but also to obtain up-to-date information about the cutting process [36]. Process control [37] provides control data for parts between cutting cycles, in order that detected machining errors can be used to predict errors for subsequent parts and to perform process corrections.

## 3. Results and Discussion

Manufacturing a product indicates the processing of raw materials and semi-finished products to obtain parts, and the assembly of these machined parts by adding manufactured items and purchased parts to complete the product. Each part must conform to specifications to fulfill its function. This can be tested by measuring and comparing the measurement result with specifications and tolerances.

A good manufacturing system is a system that produces output as predicted. This indicates that knowing the model of the cutting process and its behavior over time, we can determine the probability of producing the assumed value of the physical quantity of the workpiece. This view of the manufacturing system is not very realistic, as manufacturing systems are complex and the process model is influenced by many factors. Therefore, implementation involves several stages. In the first stage, a basic model is created, often supported by an experiment, which determines the cutting parameters in a given operating situation. The model can be expanded using various techniques for examining changes in the manufacturing system, examining disruptions in the cutting process, and adjusting the cutting parameters to the changes that have occurred. In this second stage, the implementation of corrections may be very extensive. Nevertheless, the production system functions on the basis of keeping pace with the constantly appearing disturbances of the original model.

The situation in which quality is designed is completely different. The third stage breaks away from the previous ones and creates an inverse model of the alternatives to obtain the assumed quality with a certain probability. The implementation of corrections in the process is not a follow-up task, but takes place in accordance with the probability of obtaining satisfactory quality in terms of a measurand of the workpiece.

### 3.1. Stability of the Machining System and Corrections in the Process

**Figure 3:**
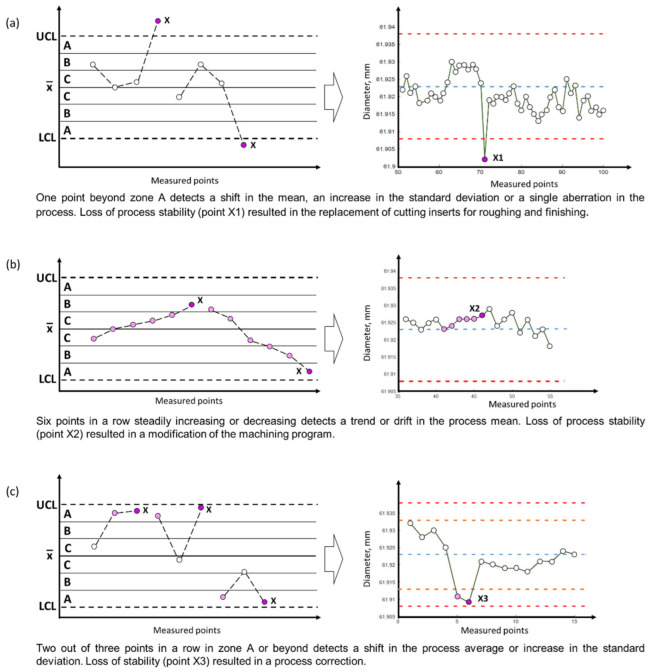

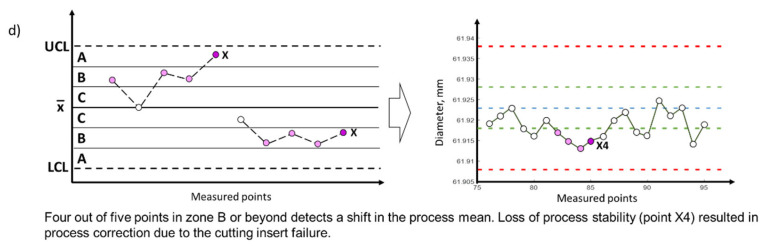
Examples of control chart test for special case variation applied to diameter measurement data at point A on the workpiece: UCL—upper control limit, $\bar{x}$— center line, LCL—lower control limit. A—zone between lines calculated at 2σ and 3σ above the center line, B—zone between lines calculated at σ and 2σ above the center line, C—zone between the center line and the line calculated at σ above the center line.

The basic requirement for a machining system operating under a certain degree of autonomy is to ensure stable operation and self-optimization in order to achieve product production within the specification. The control chart tests are designed to investigate the variability that arises from the process itself. Control limits are developed based on process data [38]. Figure 3 shows examples of control charts with the emphasis on model situations requiring intervention in the process, along with examples from the actual production process. The diameter measurement at point A for successive workpieces varies between an upper control limit and a lower control limit. Figure 3a shows an example where the measured value exceeds a lower control limit. A single disturbance for item 71 (measurement point 71) resulted in a tool change for roughing and finishing. After replacement of the tools, the values of the measured diameters for the next workpieces are within the given limits.

Complete process analysis based on the diameter measurement data was concerned with recognizing systematic or nonrandom patterns in an average value control chart and identifying the source of this process variation. The example pattern in Figure 3b relates to six consecutive workpieces with increasing diameter values.

When the Statistical Process Control (SPC) detects a pattern, it reveals that the process may be unstable. The system’s reaction depends on the detected pattern and the process itself. In the case of the pattern in Figure 3b, the machining program was modified.

For other patterns—for example, two of the three workpieces have a diameter in zone A (Figure 3c), or four of the five workpieces have a diameter in zone B or beyond (Figure 3d)—the correction was to modify the nominal depth of cut in the next pass. Since in practice the values of the machined parameters are entered to obtain the desired diameters in the workpiece program, the diameter values in the compensation program can be obtained by appropriately programming the corrections.

The cutting tests were performed for a stable thermal state of the machine tool, in order that it was possible to discount the changes in workpiece errors caused by thermal deformations of the machine tool. The process capability was assessed prior to the start of series production in accordance with the standards, as described, for example, in [39]. Measurement system capability indices were as follows:

- R&R at a level of 29.48—an acceptable system, but requiring improvement;
- SPC results—required level of Cp = 1.33, with corrections Cp = 1.46, Cpk = 1.26 (measurement at point A on the workpiece).

The main sources of diameter inaccuracies were machining process errors, such as tool wear, tool deflection, and workpiece deflection due to the cutting force. The workpiece diameter was measured; next, the machining errors for the workpiece diameter were calculated and the nominal diameters for the next workpiece were modified. The workpiece temperature was found to be variable and considerably higher than 23 °C. Therefore, different tolerance limits were assumed for workpieces immediately after machining.

**Figure 4:**
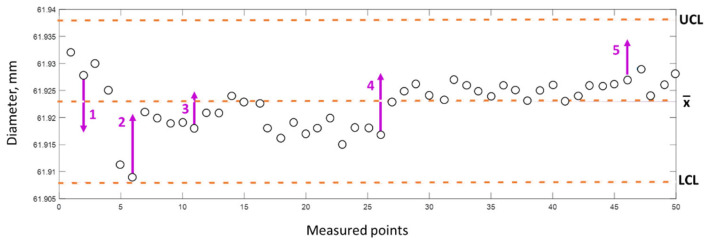
Diameter measurement value at point A on the workpiece for the first fifty workpieces (50 measured points) with process corrections marked.

Let us analyze the entire experiment by examining the capability of the process (Figure 4). We start by assigning five consecutive measurements between the pre-check limits. After this condition is met, samples are taken periodically and the stability of the process is checked.

The first measurement performed after the cutting process showed a temperature of 63 °C, and the measured values of the diameter for points A and B were within the assumed tolerance limit. However, due to the fact that this value was significant, a correction of −0.01 mm was introduced.

The measurement for SPC is carried out immediately after machining, and despite the stabilization of the working temperature of the machining system, the temperature of the workpiece varies between 40 and 90 °C. Figure 4 shows the control chart (for point A) with marked process corrections for the first 50 measured parts after the process of stabilization. Figure 4 shows a situation where the measurement data show the usefulness of a process correction applied to stabilize the process.

### 3.2. Identification of the Workpiece Temperature Compensation Model

**Figure 5:**
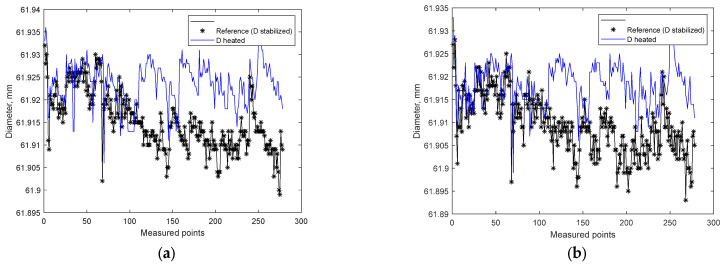
Measured values of diameter: (**a**) data gathered at point A on the workpiece; (**b**) data gathered at point B on the workpiece.

Corrections in the process were carried out in accordance with a program covering the progressive wear of the tools and the possible loss of stability. The automatic process correction system read the measurement data from the automatic measurement unit and assessed the probability of achieving the desired quality of the workpiece. The measurement used as a basis for estimating the probability came directly from the measured workpiece, while it was in a heated state. The influence of the temperature of the workpiece on the diameter value results directly from the thermal expansion of the material from which the workpiece is made. Figure 5 shows the measurement results for a heated workpiece and after stabilization. As can be seen, the results do not coincide.

**Figure 6:**
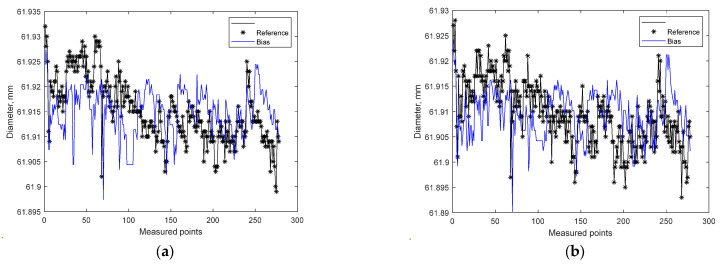
Results of estimation with the use of a model with bias: (**a**) measurement at point A on the workpiece: D_stabilized_ = −0.0086 + D_heated_; (**b**) measurement at point B on the workpiece: D_stabilized_ = −0.0088 + D_heated_.

If the difference in the measured diameter between the heated workpiece and the stabilized workpiece were influenced only by temperature, the model in this case would be constant. Goodness-of-fit was determined using a normalized root-mean-squared error [40]:

(1) $$\mathrm{fit}\left(i\right)=\frac{||x_{\mathrm{ref}}\left(:,i\right)-x\left(:,i\right)||}{||x_{\mathrm{ref}}\left(:,i\right)-\mathrm{mean}\left(x_{\mathrm{ref}}\left(:,i\right)\right)||}$$

where ‖ indicates the 2-norm of a vector, fit is a row vector of length N, and i = 1, …, N, where N is the number of channels. A model with a bias was defined, and its parameters were identified (Figure 6). The goodness-of-fit for measurement at point A on the workpiece was NRMSE = −13.41%, and for measurement at point B on the workpiece: NRMSE = −15.41%.

**Figure 7:**
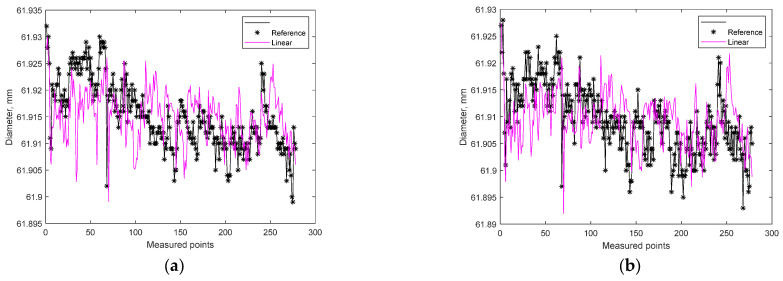
Results of estimation with the use of a linear model: (**a**) measurement at point A on the workpiece: D_stabilized_ = −0.0003 × temperature + 0.0133 + D_heated_; (**b**) measurement at point B on the workpiece: D_stabilized_ = −0.0003 × temperature + 0.0135 + D_heated_.

In order to take full account of the influence of temperature, a linear relationship of temperature compensation was obtained. In this case, the goodness-of-fit for diameter measurement at point A on the workpiece was NRMSE = −5.24%, and for measurement at point B on the workpiece: NRMSE = −7.32%. Therefore, the linear model was a better match. In Figure 7, the results of the fit are shown.

The model with a constant bias value and the linear model were developed under the assumption that each of the measurements is independent. In the first model, with a constant bias, it was indicated that the difference in the measured diameter between heated and stabilized workpieces is a constant value, and for the automatic operation of the machining system, with no breakdowns and downtimes, the system is in constant thermal conditions. For this reason, it is reasonable to ignore the temperature, which should have a similar value for each workpiece, depending only on the thermal effect of the cutting tools.

On the other hand, the linear model refers to a situation in which the temperature of the workpiece changes within a defined range immediately after machining, and the temperature is continuously measured with each workpiece. Fluctuations are due to the replacement of cutting tools and other factors. Compared with the model with a fixed value, the linear model showed a better fit with the results of the experiment, but still deviated from the reference data.

When analyzing the cutting process and subsequent workpieces, attention was drawn to the fact that while the machining of each workpiece is performed independently, it is the result of the same process.

When obtaining the values of corrections, it was decided to test the assumption that the machining system has inertia that affects subsequent measurement results. Knowing the measurement data from previous items, it is possible to estimate the value of the current measurement and predict the behavior of the system for subsequent items. Therefore, the Autoregressive with Extra Input (ARX) model was proposed, since it contains information about the previous measured values, and the model tuning is performed on the basis of the data from the current measurement [41,42].

**Figure 8:**
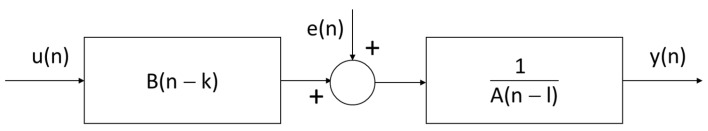
Graphical representation of the ARX model (l > k).

The ARX model structure (Figure 8) is given by the following equation:

y(n) + a_1_ y(n − 1) +...+ a_na_ y(n − n_a_) = b_1_ u(n − n_k_) +...+ b_nb_ u(n − n_b_ − n_k_ + 1) + e(n) (2)

The parameters n_a_ and n_b_ are the orders of the ARX model, and n_k_ is the delay:

- y(n)—output at measurement point n;
- n_a_ = 4—number of poles;
- n_b_ = [4 4]—number of zeros;
- n_k_ = [1 1]—number of input samples that occur before the input affects the output; also called the dead time in the system;
- y(n − 1)…y(n − n_a_)—previous outputs on which the current output depends;
- u(n − n_k_)…u(n − n_k_ − n_b_ + 1)—previous and delayed inputs on which the current output depends;
- e(n)—white-noise disturbance value.

Identification of the ARX model for measurement at point A:

A(n)y(n) = B_1_(n) u_1_(n) + B_2_(n) u_2_(n) + e(n)

A(n) = 1 − 0.64 (n − 1) − 0.12 (n − 2) − 0.1 (n − 3) − 0.05 (n − 4)

B_1_(n) = 0.07 (n − 1) + 0.03 (n − 2) − 0.09 (n − 3) + 0.07 (n − 4)

B_2_(n) = − 2.11 × 10^−5^ (n − 1) + 4.55 × 10^−6^ (n − 2) − 2.95 × 10^−5^ (n − 3) + 6.88 × 10^−6^ (n − 4)

Identification of the ARX model for measurement at point B:

A(n)y(n) = B_1_(n) u_1_(n) + B_2_(n) u_2_(n) + e(n)

A(n) = 1− 0.46 (n − 1) − 0.27 (n − 2) − 0.19 (n − 3) + 0.03 (n − 4)

B_1_(n) = 0.08 (n − 1) + 0.04 (n − 2) − 0.06 (n − 3) + 0.04 (n − 4)

B_2_(n) = − 3.37 × 10^−5^ (n − 1) − 1.71 × 10^−6^ (n − 2) + 2.01 × 10^−5^ (n − 3) − 2.27 × 10^−6^ (n − 4)

**Figure 9:**
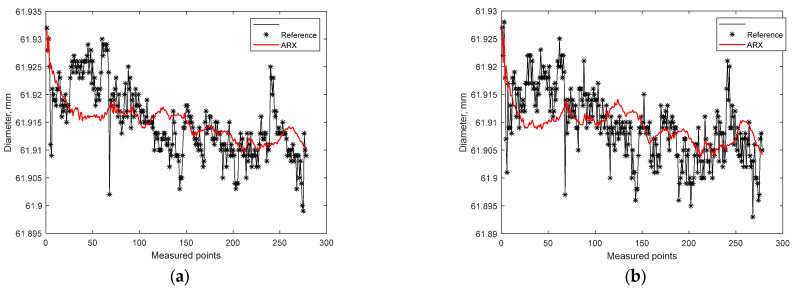
Results of estimation with the use of the ARX model: (**a**) measurement at point A on the workpiece; (**b**) measurement at point B on the workpiece.

The results of using the ARX model showed a better fit than for the linear model and the model with a constant value (Figure 9). In this case, the goodness-of-fit for measurement at point A on the workpiece was NRMSE = 14.74%, and for measurement at point B on the workpiece: NRMSE = 7.743%. Therefore, the hypothesis that the machining system has inertia was confirmed, and the value of the current measurement in undisturbed machining conditions can be estimated on the basis of measurements from previous workpieces and current information about the temperature and diameter of the heated workpiece.

**Figure 10:**
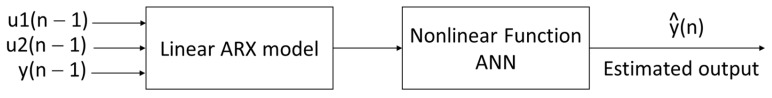
Graphical representation of the NLARX model.

**Figure 11:**
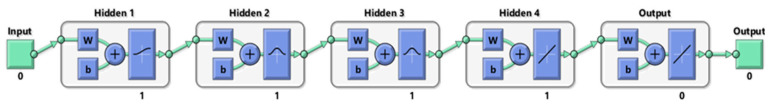
Graphical representation of ANN.

The relationship between the measured diameter for a heated workpiece and a temperature-stabilized workpiece is nonlinear. The hypothesis of nonlinearity was tested, and the test showed the existence of nonlinearity of data in the tested relationship [43]. Therefore, a model combining the ARX model with a nonlinear function in the form of an artificial neural network was used—the Nonlinear Autoregressive with Extra Input (NLARX) model (Figure 10). The structure of the estimator is described by the Wiener model, consisting of a linear regressor in the form of an ARX model with the structure described in the previous stage of the research and a nonlinear function. Data from the ARX estimator are entered into the nonlinear function. As a nonlinear function, an artificial neural network was used, which is a universal nonlinear approximator. This type of network is widely used, and there are studies confirming their usefulness and effectiveness in modeling nonlinear relationships [44]. The neural network was designed with the structure of a unidirectional perceptron with four hidden layers, one neuron in each (Figure 11). The transition functions are a log-sigmoid transfer function, a normalized radial basis transfer function, a normalized radial basis transfer function, and a linear transfer function.

**Figure 12:**
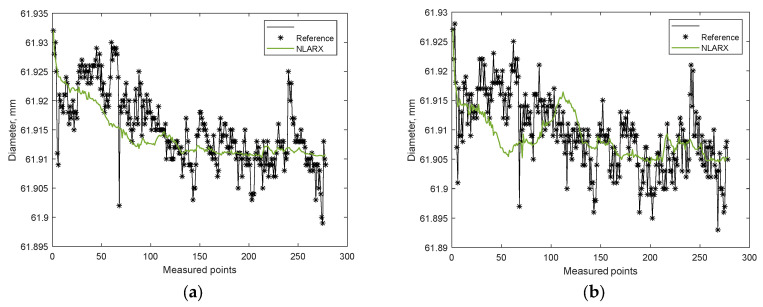
Results of estimation with the use of the NLARX model: (**a**) measurement at point A on the workpiece; (**b**) measurement at point B on the workpiece.

The results of using the nonlinear NLARX estimator confirmed its significantly better properties compared with the previously used estimators (Figure 12). Both for measurement at point A and for measurement at point B, the goodness-of-fit was higher. In this case, the goodness-of-fit for measurement at point A on the workpiece was NRMSE = 23.64%, and for measurement at point B on the workpiece: NRMSE = 9.996%.

The neural network was used to find the relationship between the measured diameter of the heated workpiece after the cutting process and the measured diameter of the stabilized workpiece. A neural network is used to fit nonlinear data. The number of layers and neurons was chosen to model the nonlinearity involved in the heating and cooling of the workpiece, observing regression plots and performance. A multi-layer back propagation algorithm was used to develop the model. The architecture of the neural network is shown in Figure 11.

Automatic process correction automatically measures the part diameter at point A and at point B on the workpiece and the temperature at the face of the part. On the basis of the measured temperature and the diameter at point A on the workpiece, the estimated value of the diameter at point A is determined for the workpiece after stabilization. Similarly, the value of the diameter at point B on the workpiece is estimated. The use of a neural network made it possible to fine-tune the model and to take account of nonlinearity.

**Figure 13:**
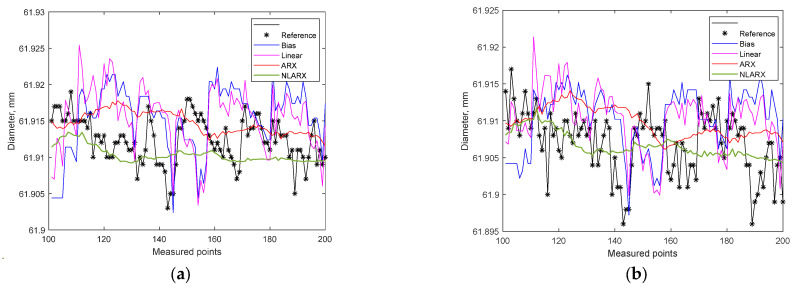
Comparison of estimation results for a part of the data (100 measured workpieces): (**a**) measurement at point A on the workpiece; (**b**) measurement at point B on the workpiece.

Table 2 summarizes the values of goodness-of-fit, and Figure 13 shows the results of identification of individual models for 100 measurements. The results of fitting different models to the experimental data indicate unambiguously that the NLARX model achieves the best fit.

Furthermore, analysis of the residuals from the developed models confirmed that the use of NLARX models provides the best reflection of the course of the process. The whiteness test for residuals is used to determine the covariance estimate:

(3) $${\hat{R}}_{\varepsilon }^{N}\left(\tau \right)=\frac{1}{N}\overset{N-\tau }{\underset{t=1}{\sum }}\varepsilon \left(t\right)\varepsilon \left(t+\tau \right)$$

If $\left\{\varepsilon \left(t\right)\right\}$ is indeed a white-noise sequence, then

(4) $$\rho_{N,M}=\frac{N}{{\hat{R}}_{\varepsilon }{\left(0\right)}^{2}}\overset{M}{\underset{\tau =1}{\sum }}{\left({\hat{R}}_{\varepsilon }^{N}\left(\tau \right)\right)}^{2}$$

would be asymptotically $\chi^{2}\left(M\right)$ distributed. Therefore, independence between the residuals $\left\{\varepsilon \left(t\right)\right\}$ can be tested by checking whether $\rho_{N,M}<\chi_{\alpha }^{2}\left(M\right)$, the α level of the $\chi^{2}\left(M\right)$ distribution [45].

The model selection criterion based on the whiteness test was dependent on checking how many of the residuals contained useful information [46]. The method was based on the assumption that if the residuals contain less information, this justifies that the model is better suited to the data. The numbers of points which do not lie in the confidence interval for various models are presented in Table 3.

The numbers of elements outside the confidence interval made it possible to confirm the usefulness of the NLARX model in process identification, due to the fact that it contains linear and nonlinear functions that act on the model regressors to produce a model result and a constant offset for that result.

## 4. Conclusions

The process correction system described here is built on the basis of an automatic measurement unit, which enables the measurement of the value of a physical quantity of the workpiece (workpiece diameter) on the production line immediately after the completion of machining. The measurement of the workpiece is subject to an error resulting from the measurement conditions, but above all from the fact that it is performed for a workpiece heated after the machining processes and not yet stabilized in temperature. In this work, we attempted to identify this error, and examined four different models that differ in complexity and reflect the influence of temperature on the measured value in different ways:

1. The model with a fixed correction value (model with bias) is the simplest and easiest to identify. The model was developed on the assumption that each workpiece is produced and measured independently. The only variable was the difference between the temperature of the measured workpiece and the reference temperature. It is a model that is easy to apply and is also applicable in the industrial conditions of automated lines where the temperature of the workpiece is maintained within a narrow, defined range of variation.
2. The model of linear dependence on the temperature difference (linear model) assumes that the measurements of physical quantities for the workpieces successively collected from the production process are independent of each other. This model extends the model with a constant correction value in a way that it is not necessary to maintain a constant value of the workpiece temperature after machining. The identified model was better fitted to the data than the model with a fixed correction value. Nevertheless, the results of the research carried out in industrial conditions revealed a poor fit of this model to the actual measurement data from the process.
3. The Autoregressive with Extra Input (ARX) model was developed on the assumption that the values of the physical quantities of the workpiece are dependent on the machining system and thus show a relationship with previously manufactured items. This applied both to phenomena of tool wear type, which are progressive, and to the potential values of the process itself, such as temperature. Taking into account the inertia of the system positively influenced the goodness-of-fit of the identified ARX model to the actual data from the production process.
4. The Nonlinear Autoregressive with Extra Input (NLARX) model was developed on the assumption that the values of physical quantities of consecutive items on the production line show a nonlinear relationship. Nonlinearity for real data in industrial research has been tested and confirmed. The structure of the ARX model was developed by adding a segment in the form of an artificial neural network, whose task was to tune the model nonlinearly. The goodness-of-fit results for the identified NLARX model were confirmed by means of the normalized root-mean-squared error value and the white-noise test for residuals.

## Figures and Tables

**Table 1:** Material specification.

**Chemical Composition, Mass %**	
C	3.65 ÷ 3.80
Si	1.60 ÷ 2.10
Mn	0.40 ÷ 0.80
Cr	0.15 ÷ 0.35
Cu	0.30 ÷ 0.60
Fe	remainder
**Microstructure EN ISO 945**	
Type of matrix	Pearlitic, fine lamellar
Permissible ferrite	5% MAX, well distributed
Shape of graphite	I
Distribution of graphite	A: min 70%; D–E: <4% dispersed; B: reminder
Size of graphite	3–4–5: min 70%; 2: traces; 6: ≤15%
**Mechanical properties of casting**	
Hardness HBW on face	170–210
Tensile strength Rm, N/mm^2^	Min 170
Young’s modulus E, N/mm^2^	95,000
**Physical properties**	
Density	7.1 kg/dm^3^
Coefficient of thermal expansion at 20 °C	9 × 10^−6^ 1/K
Thermal conductivity at 100 °C	52 W/(m·K)

**Table 2:** Comparison of goodness-of-fit for different models.

Model	Goodness-of-Fit: Measurement at Point A on the Workpiece	Goodness-of-Fit: Measurement at Point B on the Workpiece
Constant bias	−13.41%	−15.41%
Linear model	−5.24%	−7.32%
Discrete-time ARX model	14.74%	7.743%
Nonlinear ARX model with 1 output and 2 inputs	23.64%	9.996%

**Table 3:** Comparison of whiteness test for residuals.

Model	Number of Elements Outside the Confidence Interval:	
	Measurement at Point A on the Workpiece	Measurement at Point B on the Workpiece
Constant bias	41	23
Linear model	14	14
Discrete-time ARX model	14	14
Nonlinear ARX model with 1 output and 2 inputs	12	12

## Data Availability

Not applicable.

## References

[1] Tambare P., Meshram C., Lee C.-C., Ramteke R.J., Imoize A.L. (2022). Performance Measurement System and Quality Management in Data-Driven Industry 4.0: A review. Sensors.

[2] Cheng Y., Wang Z., Chen X., Li Y., Li H., Li H., Wang H. (2019). Evaluation and Optimization of Task-oriented Measurement Uncertainty for Coordinate Measuring Machines Based on Geometrical Product Specifications. Appl. Sci..

[3] Flack D., Hannaford J. (2012). Good Practice Guide No. 80 Fundamental Good Practice in Dimensional Metrology.

[4] Ji Z., Li P., Zhou Y., Wang B., Zang J., Liu M. (2018). Toward New-Generation Intelligent Manufacturing. Engineering.

[5] Zawada-Tomkiewicz A., Tomkiewicz D., Majewski M., Kacalak W. (2020). Monitoring System with a Vision Smart Sensor. Innovations Induced by Research in Technical Systems.

[6] Cheng Y., Zhang X., Zhang G., Jiang W., Li B. (2022). Thermal Deformation Analysis and Compensation of the Direct-Drive Five-Axis CNC Milling Head. J. Mech. Sci. Technol..

[7] (2015). Machine Tools-Numerical Compensation of Geometric Errors.

[8] Schwenke H., Knapp W., Haitjema H., Weckenmann A., Schmitt R., Delbressine F. (2008). Geometric Error Measurement and Compensation of Machines—An update. CIRP Ann..

[9] Pimenov D.Y., Guzeev V.I., Mikolajczyk T., Patra K. (2017). A Study of the Influence of Processing Parameters and Tool Wear on Elastic Displacements of the Technological System Under Face Milling. Int. J. Adv. Manuf. Technol..

[10] Richardson D.J., Keavey M.A., Dailami F. (2006). Modelling of Cutting Induced Workpiece Temperatures for Dry Milling. Int. J. Mach. Tools Manuf..

[11] Lin S., Peng F., Wen J., Liu Y., Yan R. (2013). An Investigation of Workpiece Temperature Variation in End Milling Considering Flank Rubbing Effect. Int. J. Mach. Tools Manuf..

[12] (2003). Geometrical Product Specifications (GPS)—Systematic Errors and Contributions to Measurement Uncertainty of Length Measurement Due to Thermal Influences.

[13] Groos L., Held C., Keller F., Wendt K. (2014). Good Practice Guide for Assessing the Fitness for Purpose for Dimensional Measurements on Machine Tools.

[14] Li Y., Yu M., Bai Y., Hou Z., Wu W. (2021). A Review of Thermal Error Modeling Methods for Machine Tools. Appl. Sci..

[15] Abele E., Fiedler U. (2004). Creating Stability Lobe Diagrams during Milling. Ann. CIRP.

[16] Altintas Y., Weck M. (2004). Chatter Stability in Metal Cutting and Grinding. Ann. CIRP.

[17] Das S.R., Nayak R.P., Dhupal D. (2012). Optimization of cutting parameters on tool wear and workpiece surface temperature in turning of AISI D2 steel. Int. J. Lean Think..

[18] Bhirud N.L., Gawande R.R. (2017). Optimization of Process Parameters During End Milling and Prediction of Work Piece Temperature Rise. Arch. Mech. Eng..

[19] De Chiffre L., González-Madruga D., Dalla Costa G., Sonne M.R., Mohammadi A., Hattel J.H., Hansen H.N., Mohaghegh K., Meftahpour M., Meinertz J. (2018). Accurate Measurements in a Production Environment Using Dynamic Length Metrology (DLM). Procedia CIRP.

[20] De Chiffre L., González-Madruga D., Sonne M.R., Dalla Costa G., Hattel J.H., Hansen H.N. (2021). Dynamic Length Metrology (DLM) For Accurate Dimensional Measurements in a Production Environment by Continuous Determination and Compensation of Thermal Expansion Effects in Turning Steel. Meas. Sci. Technol..

[21] Mutilba U., Gomez-Acedo E., Kortaberria G., Olarra A., Yagüe-Fabra J.A. (2017). Traceability of On-Machine Tool Measurement: A Review. Sensors.

[22] Simson K., Lillepea l., Hemming B., Widmaier T. Traceable In-Process Dimensional Measurement—European Metrology Research Programme, Project No. IND62. Proceedings of the 9th International DAAAM Baltic Conference Industrial Engineering.

[23] Ačko B., Klobučar R., Ačko M. (2015). Traceability of In-Process Measurement of Workpiece Geometry. Procedia Eng..

[24] O’Sullivan D., Cotterell M. (2002). Workpiece Temperature Measurement in Machining. Proc. Inst. Mech. Eng. Part B J. Eng. Manuf..

[25] Liu Z.Q. (1999). Repetitive Measurement and Compensation to Improve Workpiece Machining Accuracy. Int. J. Adv. Manuf. Technol..

[26] Yang S., Yuan J., Ni J. (1996). The Improvement of Thermal Error Modelling and Compensation on Machine Tools by Neural Network. Int. J. Mach. Tools Manuf..

[27] Budak E., Ozlu E. (2007). Analytical Modeling of Chatter Stability in Turning and Boring Operations: A Multi-Dimensional Approach. CIRP Ann..

[28] Wu H., Zhang H., Guo Q., Wang X., Yang J. (2008). Thermal Error Optimization Modelling and Re-Al-Time Compensation on a CNC Turning Centre. J. Mater. Processing Technol..

[29] Brecher C., Esser M., Witt S. (2009). Interaction of Manufacturing Process and Machine Tool. CIRP Ann..

[30] Lin Z.C., Chow J.J. (2001). Integration Planning Model of IDEF0 and STEP Product Data Representation Methods in a CMM Measuring System. Int. J. Adv. Manuf. Technol..

[31] Li X.P., Deng Y.H., Li X.Z. (2021). Application of Multisensor Information Fusion Technology in the Measurement of Dynamic Machining Errors of Computer Numerical Control (CNC) Machine Tools. J. Sens..

[32] Zawada-Tomkiewicz A., Wierucka I. (2018). A Case Study in Technological Quality Assurance of a Metric Screw Thread. Measurement.

[33] Mian S.H., Al-Ahmari A.M. (2017). Application of the Sampling Strategies in the Inspection Process. Proc. Inst. Mech. Eng. Part B J. Eng. Manuf..

[34] Horst J.A., Hedberg T.D., Feeney A.B. (2019). On-Machine Measurement Use Cases and Information for Machining Operations.

[35] Liu C., Xu X. (2017). Cyber-physical Machine Tool—The Era of Machine Tool 4.0. Procedia CIRP.

[36] Kunzmann H., Pfeifer T., Schmitt R., Schwenke H., Weckenmann A. (2005). Productive Metrology-Adding Value to Manufacture. CIRP Ann. Manuf. Technol..

[37] Bandy H.T., Donmez M.A., Gilsinn D.E., Kennedy M., Yee K.W., Ling A.V., Wilkin N.D. (2001). A Methodology for Compensating Errors Detected by Process-Intermittent Inspection.

[38] Jin X.L., Song J.B., Peng J.X., Pan X.P., Guo R., Xing X.F. (2022). Study on the Established Customized Limits for the Daily Quality Assurance Procedure. J. Radiat. Res..

[39] Gupta B.C. (2021). Process and Measurement System Capability Analysis in Statistical Quality Control: Using MINITAB, R, JMP and Python.

[40] James G., Witten D., Hastie T., Tibshirani R. (2013). An Introduction to Statistical Learning with Applications in R.

[41] Xie J., Li C., Li N., Li P., Wang X., Gao D., Yao D., Xu P., Yin G., Li F. (2021). Robust Autoregression with Exogenous Input Model for System Identification and Predicting. Electronics.

[42] Ruhm K.H. (2016). Dynamics and stability—A Proposal for Related Terms in Metrology from a Mathematical Point of View. Measurement.

[43] Schreiber T., Schmitz A. (1996). Improved Surrogate Data for Nonlinearity Tests. Phys. Rev. Lett..

[44] Schoukens J., Ljung L. (2019). Nonlinear System Identification: A User-Oriented Road Map. IEEE Control. Syst. Mag..

[45] Ljung L. (1999). System Identification: Theory for the User.

[46] Zawada-Tomkiewicz A., Storch B. (2004). Introduction to the Wavelet Analysis of a Machined Surface Profile. Adv. Manuf. Sci. Technol..

